# Projected Distributions and Diversity of Flightless Ground Beetles within the Australian Wet Tropics and Their Environmental Correlates

**DOI:** 10.1371/journal.pone.0088635

**Published:** 2014-02-20

**Authors:** Kyran M. Staunton, Simon K. A. Robson, Chris J. Burwell, April E. Reside, Stephen E. Williams

**Affiliations:** 1 Centre for Tropical Biodiversity & Climate Change and School of Marine and Tropical Biology, James Cook University, Townsville, Queensland, Australia; 2 Queensland Museum, Brisbane, Queensland, Australia; 3 Environmental Futures Centre and Griffith School of Environment, Griffith University, Brisbane, Queensland, Australia; CNRS-Montpellier, France

## Abstract

With the impending threat of climate change, greater understanding of patterns of species distributions and richness and the environmental factors driving them are required for effective conservation efforts. Species distribution models enable us to not only estimate geographic extents of species and subsequent patterns of species richness, but also generate hypotheses regarding environmental factors determining these spatial patterns. Projected changes in climate can then be used to predict future patterns of species distributions and richness. We created distribution models for most of the flightless ground beetles (Carabidae) within the Wet Tropics World Heritage Area of Australia, a major component of regionally endemic invertebrates. Forty-three species were modelled and the environmental correlates of these distributions and resultant patterns of species richness were examined. Flightless ground beetles generally inhabit upland areas characterised by stable, cool and wet environmental conditions. These distribution and richness patterns are best explained using the time-stability hypothesis as this group’s primary habitat, upland rainforest, is considered to be the most stable regional habitat. Projected changes in distributions indicate that as upward shifts in distributions occur, species currently confined to lower and drier mountain ranges will be more vulnerable to climate change impacts than those restricted to the highest and wettest mountains. Distribution models under projected future climate change suggest that there will be reductions in range size, population size and species richness under all emission scenarios. Eighty-eight per cent of species modelled are predicted to decline in population size by over 80%, for the most severe emission scenario by the year 2080. These results suggest that flightless ground beetles are among the most vulnerable taxa to climate change impacts so far investigated in the Wet Tropics World Heritage Area. These findings have dramatic implications for all other flightless insect taxa and the future biodiversity of this region.

## Introduction

Climate change is expected to negatively impact biodiversity due to increased exposure of species to deleterious climatic conditions. Many studies have raised concern that this is potentially one of the greatest threats to global biodiversity ever faced [1]–[5]. Species’ distributions have already shifted upwards in elevation and polewards in response to climate change [6], [7]. Montane fauna, especially those with limited dispersal abilities, are generally unable to migrate higher than the mountain they currently inhabit and are therefore highly threatened from climate change due to projected range contractions [8]. Temperatures are predicted to increase by 1.1–6.4°C by the end of this century [9]. With the vulnerability of dispersal-limited montane fauna to climate change, a greater understanding of the links between environmental conditions and species’ distributions and resultant biodiversity patterns is vital to conservation biology and biodiversity management.

Multiple factors drive the distributions of species and therefore patterns of biodiversity [10]. Climate is generally thought of as the primary driver of terrestrial species’ distributions [11]. Consequently, the climatic envelopes of species (the area in which suitable conditions exist, allowing a species to persist despite influences from competitors and natural enemies) have been modelled and used to project range shifts in response to future climatic impacts [1], [8], [11].

Globally, species richness increases toward the equator, and several environmental parameters, such as solar energy, productivity and physical heterogeneity, have been suggested to drive this pattern [12]. These same parameters have also been used to explain changes in species richness across elevational gradients, where, for example, lowland areas exposed to higher levels of solar energy are more productive and therefore may support more species than uplands habitats [13]. Other studies have reveal mid-elevational peaks in richness which are attributed to factors such as overlapping ranges of lowland and upland specialist fauna [14], [15]. Additionally, richness may peak at high elevations in response to favourable climatic conditions or greater resource availability [16], [17].

Often, explanations for biodiversity patterns overlook historical factors which may exert considerable influence on current assemblage structure and species richness, especially for taxa with low dispersal abilities [18]. Theories incorporating the influence of historical factors on species assemblages include the time-stability hypothesis, where habitat stability affects the rate of *in situ* evolution of species [19], and the species filtering effect, where changes in historical environmental conditions selectively drive local populations extinct resulting in patterns of species richness determined largely by the process of non-random extinction, rather than evolution [18], [20]. Whether the theories which contribute to biodiversity patterns consider current or historical influences, it is important to note that changes in biodiversity are most likely caused by a variety of factors rather than simply one mechanism [21].

The tropical rainforests of the Wet Tropics World Heritage Area of Australia (hereafter the “Wet Tropics”) boast among the highest levels of biodiversity within Australia [22]. The mean saturated adiabatic lapse rate of temperature within the rainforest of the Wet Tropics is approximately 1°C per 200 m in altitude [23]. Therefore, species within this area would be expected to shift their distributions upwards by 220–1280 m by the year 2100. Clearly, distributional shifts of this magnitude are likely to have substantial impacts on assemblage composition and biodiversity. In fact, Williams *et al*. (2003) predicted catastrophic declines in regionally-endemic vertebrates within the Wet Tropics rainforests. Whilst much effort has been devoted to investigating the drivers of biodiversity of *vertebrates* within the Wet Tropics [24], the vast majority of work concerning *invertebrates* has been taxonomic in nature and links between biodiversity of these taxa and environmental data remain to be comprehensively established. Invertebrate species account for 75% of biodiversity worldwide [25] and are responsible for a multitude of vital ecosystem functions [26]. Understanding environmental factors driving invertebrate distributions and richness within the Wet Tropics is, therefore, crucial to determining the ecological impacts of climate change in this region.

Among the invertebrate fauna of the Wet Topics region, flightless insects, particularly Coleoptera and Hemiptera, have been relatively well studied. Of these flightless insects, 50% of a group (containing 274 species) studied by Yeates *et al.* (2002) were endemic to single subregions (geographically distinct upland forest blocks). This is a high proportion when compared to 15% of vertebrates endemic to single subregions in rainforests of the Wet Tropics [20], [27]. A diverse group of flightless insects within the Wet Tropics is predatory beetles from the family Carabidae, commonly known as ground beetles. Ground beetles are well-known and highly diverse with approximately 40,000 described species globally [28]. Carabid beetles can be divided into three main habitat-specific groups; those associated with the ground (the vast majority of species), arboreal or aquatic habitats [29], [30]. The Australian carabid fauna contains an abnormally high proportion (∼45%) of species with atrophied wings [31] and furthermore, about 75% of ground-associated species are flightless [31]. As ground beetles are generally predatory, feeding on other leaf litter invertebrates, they form an important functional group in forest-floor habitats [32].

While the taxonomy of flightless ground beetles within the Wet Tropics is well known, few studies have addressed the environmental factors affecting their distributions and diversity. This study utilises a species distribution modelling approach to elucidate environmental factors that most significantly contribute to explaining observed spatial patterns of species distributions and richness of flightless ground beetles. We then use species distribution models, in combination with projected emission scenarios in the region, to predict the impacts of future climatic change on the distributions and diversity of this important group.

## Methods

### Study Area

**Figure 1:**
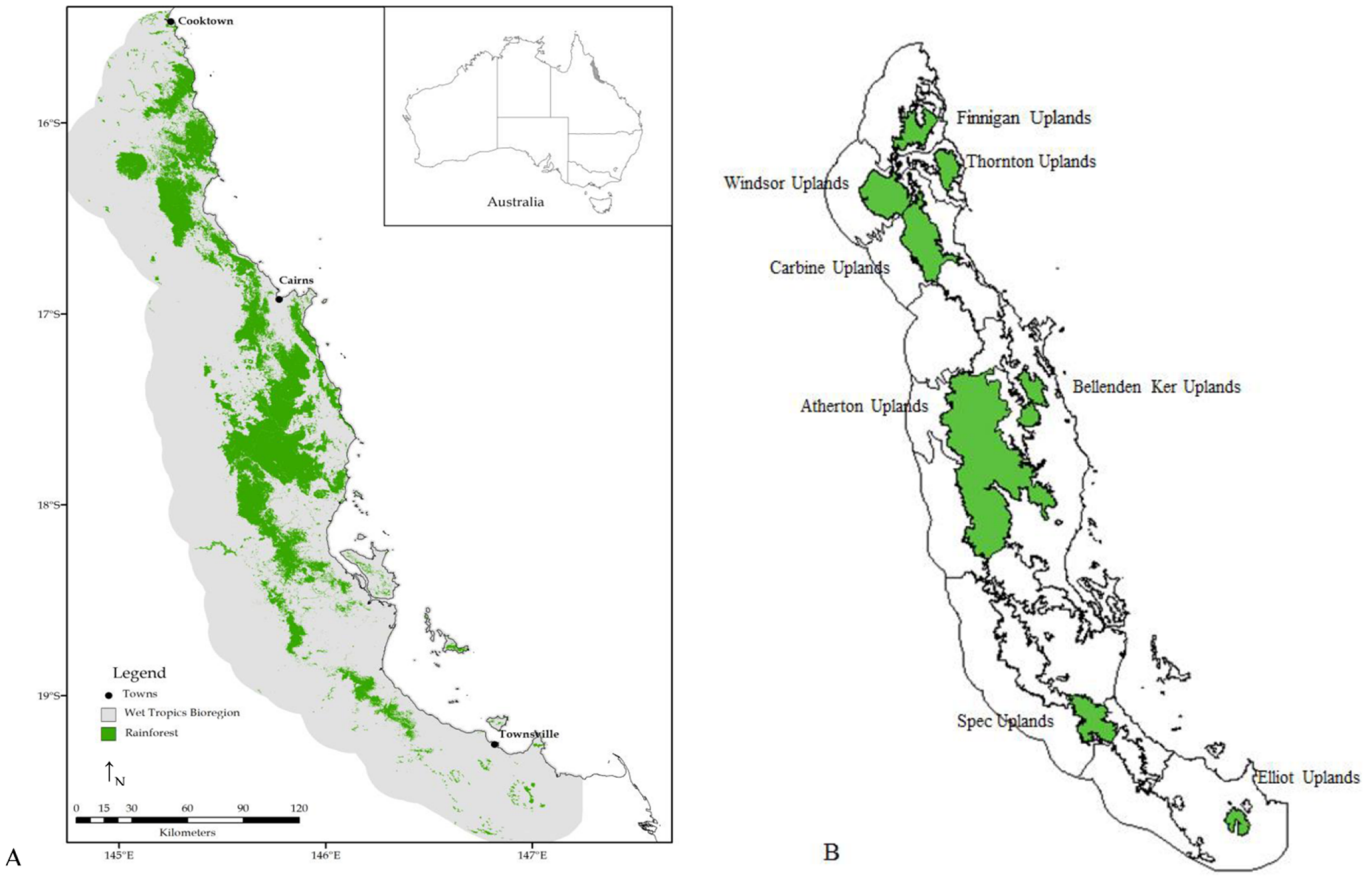
Map of the Wet Tropics bioregion. **A.** showing the current extent of rainforest, **B.** highlighting subregions specifically discussed in this paper.

This study was conducted in north-eastern Australia (20° to 15°S and 147° to 145°E) within the Wet Tropics bioregion which is approximately 10,000 km^2^ in area (Figure 1). The Wet Tropics has been listed as a World Heritage Area since 1988 due to the high biodiversity and endemism of the region’s rainforests. This study was confined to rainforest and covered a range of structural rainforest types across elevational gradients from complex mesophyll vine forest in the fertile lowlands to upland simple notophyll vine forest and, in the case of Bellenden Ker Uplands, simple microphyll vine-fern thickets above 1,500 m a.s.l. [33].

Generally, annual rainfall throughout the Wet Tropics is high (2,000–8,000 mm per year) [34] with approximately 75–90% of precipitation falling between November and April [35]. Additionally, rainforests located at elevations above 1,000 m a.s.l. receive up to 66% of their monthly water input from cloud stripping [36]. Rainfall estimates, therefore, under-estimate total water input. Approximately one-third of the Wet Tropics bioregion is higher than 600 m a.s.l., where annual mean temperatures are below 22°C [37].

### Ethics Statement

Many locality records used in this study derive from specimens collected on Mt Spec, Atherton Uplands including Wooroonooran National Park, Mt Bellenden Ker, Mt Lewis and Mt Windsor under permit WITK05468508 provided by the Queensland Government Department of Environment, Resource and Minerals. All other locality data were derived from specimens in the collections of the Queensland Museum.

### Species Data

Carabid beetle species data consisted of 949 geographically unique records of 43 flightless species. Locality records of species were primarily accessed from the specimen database of the Queensland Museum and additional data, obtained from specimens collected during this study, are lodged with, and available from the Centre for Tropical Biodiversity and Climate Change. These data were used to model the distributions of the 43 species within the Wet Tropics region. The number of data points varied considerably among species. The most commonly recorded species was *Castelnaudia obscuripennis* with 92 point localities while the least common was *Pamborus elegans* with only three records confirmed by the Queensland Museum. While using models derived from only three point localities is not ideal, expert inspection of the model deemed it a good representation of the extremely restricted distribution of this species and therefore warranted its use.

### Environmental Data

Bioclimatic variables used to model carabid distributions were annual mean temperature (BC01), temperature seasonality (BC04), maximum temperature of warmest period (BC05), minimum temperature of coldest period (BC06), annual precipitation (BC12), precipitation seasonality (BC15), precipitation of wettest quarter (BC16) and precipitation of driest quarter (BC17). These variables have been strongly linked to vertebrate and dipteran distributions in the Wet Tropics [38]–[40]. These climate data were used as the baseline climate, defined as a 30 year average centred around 1990 (between 1976 and 2005). The bioclimatic variables were derived using the Anuclim 5.1 software [41] and a 80-m-resolution digital elevation model (DEM; resampled from GEODATA 9-second DEM, ver.2; Geoscience Australia, http://www.ga.gov.au/).

Vegetation data used were the National Vegetation Information System broad vegetation subgroups (Australian Government Department of the Environment & Water Resources, 2004) at a resolution of 250 m. These data comprise 32 major vegetation types throughout the Wet Tropics. The Wet Tropics region was delineated into subregions as defined in detail in Williams et al. [42] (Figure 1B).

### Species Distribution Models

Species distributions models (SDMs) were generated using Maxent, a maximum entropy algorithm (Maxent v. 3.3.3; [46]). Default settings were used [43]. Maxent is a machine learning modelling technique which utilises the concept of maximum entropy to model species distributions [44], [45]. The explanatory variables in each of the models were the eight bioclimatic variables and 32 vegetation types. As Maxent automatically regulates effects of correlated variables, only ecologically insignificant variables need to be removed [46]. The resulting potential distributions were clipped by subregions within which species are known to be present (from the occurrence records) to reduce overestimations of species distributions [47]. These realised distributions were reviewed by the expert on this insect group within this region (Geoff Monteith) and incongruous points were removed and the models rerun or inadequate models removed completely. All models presented had area under the receiver-operating characteristic curve (AUC) values greater than 0.9 and are therefore considered to perform excellently [48]. The use of the AUC metric has been criticised regarding concerns such as its: equal treatment of commission and omission errors, spatial independence and relationship to the spatial extent investigated [49]–[51]. However, Jiménez-Valverde [49] state that all of these concerns are common to any discrimination measure used in this context, not just the AUC. Furthermore, Santika [52] found that the AUC metric correctly identifies a model’s ability to successfully capture the dominant environmental determinants. This ability of the AUC metric to successfully capture dominant environmental determinants, in combination with opinions of the expert reviewer, makes it useful in this study.

Future species distributions were generated by projecting each species model onto future climate scenarios [45]. For this model run of baseline and future projections, the same eight bioclimatic variables were used; however, vegetation type was excluded. Using vegetation type in future models is beyond the scope of this project for several reasons: 1) no future projections of vegetation type are available for use in modelling, 2) using modelled future vegetation projections would substantially increase the uncertainty of the projected species distributions, and 3) climate is a good proxy for future vegetation [53]. The climate for 2080 was based on a 30 years average between 2066 and 2095. Future climate surfaces were derived from the Intergovernmental Panel on Climate Change’s Special Report on Emissions Scenarios (SRES) three scenarios of B1, A1B, and A2 [54]. Projections of global greenhouse gas emissions, and subsequent global temperatures, increase progressively with each scenario from B1 to A1B to A2 [55]. Eight global circulation models (GCMs), from the IPPC fourth assessment report [56], were used to create future climate layers including: BCCR-BCM 2.0, CSIRO-Mk 3.0, CSIRO-Mk 3.5, GISS AOM, INM CM 3.0, MIROC 3.2 (hires), MIROC 3.2 (medres) & NCAR CCSM 3.0. Effects of each model on determining future climate layers were weighted to account for unequal numbers of realisations between GCMs and years [57]. Also, similar to Reside *et al.* (2012), mean projections for each year and scenario were obtained by initially summarising the multiple realisations within GCM and then across GCMs.

Multivariate analyses utilised additional environmental data considered most relevant to the ecology of these carabids including slope, aspect, distance to stream and foliage projected cover. Spatial surfaces of slope and aspect were derived from a 250 m digital elevational model obtained from Geoscience Australia (resampled from GEODATA 9S DEM Version 2; Geoscience Australia, http://www.ga.gov.au/). Both slope and aspect surfaces were created using the *r.slope* command in the GRASS package from the R statistical program v2.12.1 [58], [59]. The surface of distance to stream (ln(distance+1)), was built using Spatial Analyst in ESRI ARCGIS and maps from Geoscience Australia’s Global Map Australia 1 M 2001 product (http://www.ga.gov.au/nmd/products/digidat/1m.htm). Foliage projected cover was obtained from the Queensland Department of Natural Resources and Water over a 30-year average at 250 m resolution [59].

### Statistical Analyses

A species richness layer was created as an ASCII file so that the environmental variables best correlated with changes in richness could be determined. Firstly, distribution models for each species were converted to a binary format. To do this the areas with a probability of presence either equal to or greater than a threshold value that minimizes 6 *training omission rate +0.04 *cumulative threshold +1.6 *fractional predicted area, were set to one (suitable). All other areas within the models were set to zero (unsuitable). This threshold has been determined to produce the most realistic distributions by experts concerning species within this region [42]. All 43 species distribution models were then summed and each 80 m by 80 m cell was represented by a cumulative value.

We randomly extracted 10,000 points from the species richness layer and used best sub-set multiple linear regression to analyse correlations between richness values and co-located environmental data (bioclimatic, vegetation and other environmental factors) using the *leaps* package from the R statistical program v2.12.1 [58]. The best models derived from any combination of the explanatory variables were determined using Bayesian Information Criterions (BICs). This statistical modelling technique differs slightly from the more standard variable selection methods of stepwise regression, as it better accounts for co-linearity problems between explanatory variables [60]. However, some removal of significantly correlated variables was still required. The final model selected used four environmental factors as addition of a fifth produced only marginal improvements in the BIC (−9490 for four factors, −9541 for five factors). To visualise the significant relationships, species richness and environmental factor residuals from this model were plotted using the *car* package from the R statistical program v2.12.1 [58].

The species richness layer was also weighted by endemism to visually assess which areas contained higher proportions of species with restricted ranges. Rather than using a value of one to indicate presence of a species for a given cell when overlaying individual SDMs to create a species richness model, the inverse of each species’ area of range size, derived from Maxent output, was used. Therefore subregions containing a high proportion of species displaying restricted ranges expressed comparatively higher values than subregions dominated by species with larger ranges.

Projected changes in the proportion of total abundance of each species were calculated using the approach described in VanDerWal *et al*. (2009b). This approach assumes a relationship between environmental suitability and a species’ abundance. Environmental suitability was derived from the probability of presence value provided by the Maxent species distribution model output. Summed environmental suitability across a species’ distribution can be used as an index of total population size [39]. Therefore, proportional changes in total abundance can be estimated from changes in environmental suitability derived from the projected future model output. Proportional changes in total abundance for each species were averaged for each model output per decade. These resulting decadal averages for all 43 species were then averaged again to give an estimate for the entire group, per emission scenario outlined above.

Best sub-set multiple linear regression was used to determine which combination of bioclimatic variables best explained variation in the proportional changes in total abundance of the 14 most vulnerable (those projected to lose >50% of current population) subregional endemic species (for simplicity, only results from the year 2080 and SRES A2 are shown). This analysis used the *leaps* package from the R statistical program v2.12.1 [58]. A Fisher test, using the *fisher.test* function in the R statistical program v2.12.1 [58], was also run to determine if there was a positive association between occupation of drier habitats and higher reduction in proportional abundance for all 16 subregional endemics (year 2080, SRES A2). Species projected to lose more of their proportional abundance by 2080 were inferred to be at higher risk of extinction than those projected to maintain more of their abundance.

## Results

**Figure 2:**
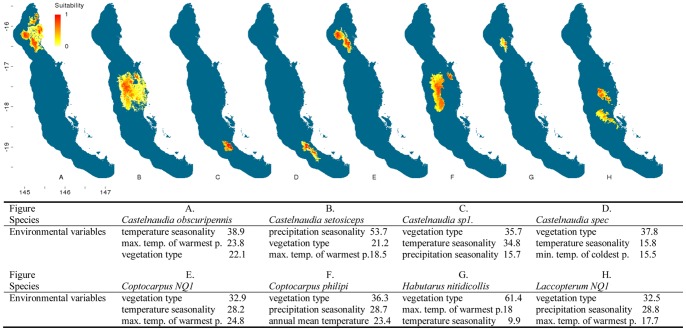
Distribution models of flightless ground beetle species in the Wet Tropics bioregion. Noted are the three highest-ranked environmental correlates selected by Maxent with percentage contribution of each variable to the model. Max. temp. of warmest p. = maximum temperature of the warmest period, min. temp. of coldest p. = minimum temperature of the coldest period. Habitat suitability for each species is indicated in a gradient from yellow (less suitable) to red (highly suitable). Blue areas are considered unsuitable for the species.

**Figure 3:**
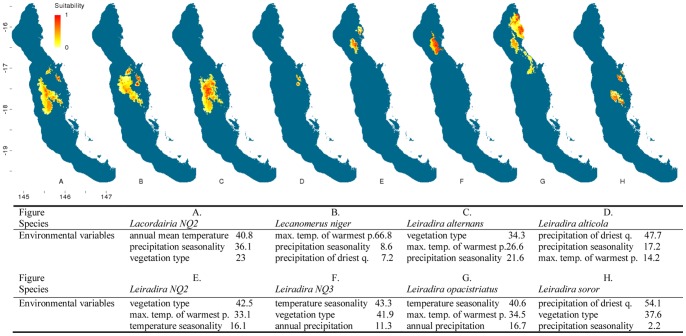
Distribution models of flightless ground beetle species in the Wet Tropics bioregion. Noted are the three highest-ranked environmental correlates selected by Maxent with percentage contribution of each variable to the model. Max. temp. of warmest p. = maximum temperature of the warmest period, min. temp. of coldest p. = minimum temperature of the coldest period. Habitat suitability for each species is indicated in a gradient from yellow (less suitable) to red (highly suitable). Blue areas are considered unsuitable for the species.

**Figure 4:**
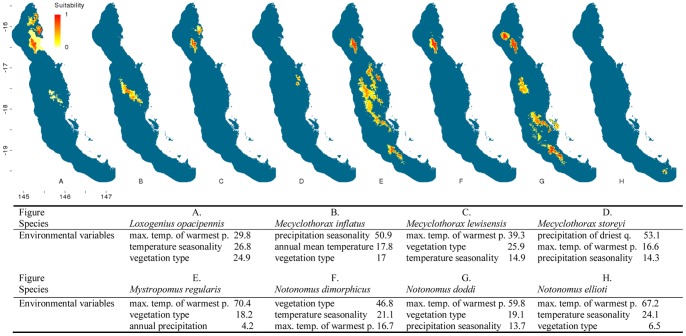
Distribution models of flightless ground beetle species in the Wet Tropics bioregion. Noted are the three highest-ranked environmental correlates selected by Maxent with percentage contribution of each variable to the model. Max. temp. of warmest p. = maximum temperature of the warmest period, min. temp. of coldest p. = minimum temperature of the coldest period. Habitat suitability for each species is indicated in a gradient from yellow (less suitable) to red (highly suitable). Blue areas are considered unsuitable for the species.

**Figure 5:**
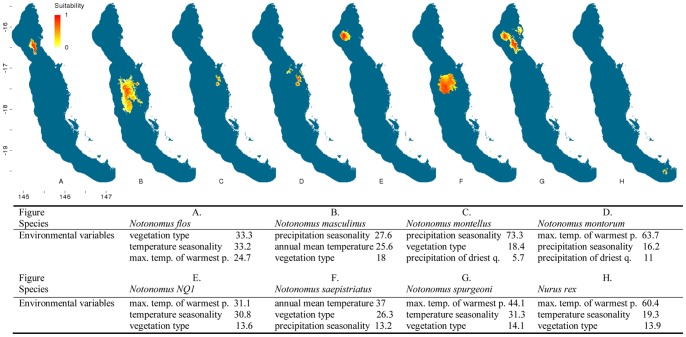
Distribution models of flightless ground beetle species in the Wet Tropics bioregion. Noted are the three highest-ranked environmental correlates selected by Maxent with percentage contribution of each variable to the model. Max. temp. of warmest p. = maximum temperature of the warmest period, min. temp. of coldest p. = minimum temperature of the coldest period. Habitat suitability for each species is indicated in a gradient from yellow (less suitable) to red (highly suitable). Blue areas are considered unsuitable for the species.

**Figure 6:**
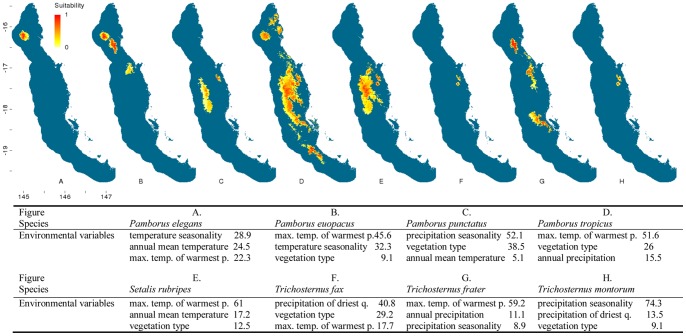
Distribution models of flightless ground beetle species in the Wet Tropics bioregion. Noted are the three highest-ranked environmental correlates selected by Maxent with percentage contribution of each variable to the model. Max. temp. of warmest p. = maximum temperature of the warmest period, min. temp. of coldest p. = minimum temperature of the coldest period. Habitat suitability for each species is indicated in a gradient from yellow (less suitable) to red (highly suitable). Blue areas are considered unsuitable for the species.

**Figure 7:**
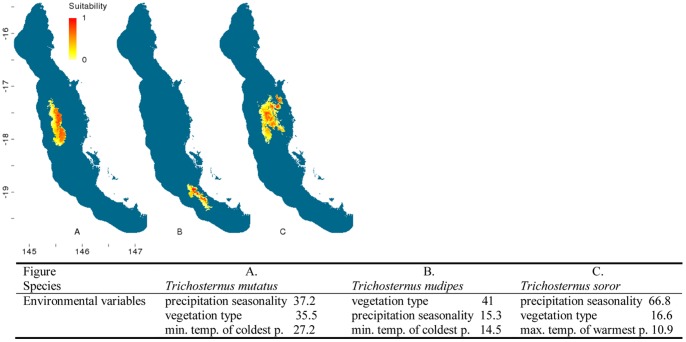
Distribution models of flightless ground beetle species in the Wet Tropics bioregion. Noted are the three highest-ranked environmental correlates selected by Maxent with percentage contribution of each variable to the model. Max. temp. of warmest p. = maximum temperature of the warmest period, min. temp. of coldest p. = minimum temperature of the coldest period. Habitat suitability for each species is indicated in a gradient from yellow (less suitable) to red (highly suitable). Blue areas are considered unsuitable for the species.

The distribution modelling outputs for the flightless ground beetles highlighted that almost half (20) of the 43 species are predicted to be distributed solely within the central Wet Tropics (Figures 2–7). Of these centrally located species five are predicted to exclusively occur within Bellenden Ker Uplands while a further 12 species had broader distributions extending into adjacent mountain ranges. Thirteen species are predicted to be restricted to the northern subregions with only small southern extensions of distributions predicted over the Black Mountain Barrier for *Leiradira opacistriatus* and *Pamborus euopacus*. Two northern species, *Notonomus NQ1* and *Pamborus elegans*, are endemic to Windsor Uplands, a western mountain range situated in the rain shadow of the Carbine Uplands. Of the five southern species modelled, the highly restricted *Nurus rex* and *Notonomus ellioti* were only found in Mt Elliot, the most southern mountain in the Wet Tropics bioregion. Finally, five species, each from different genera, had widespread predicted distributions throughout the Wet Tropics with *Pamborus tropicus* displaying the greatest range of all flightless ground beetles examined, inhabiting such diverse sub-regions as Windsor Uplands, Bellenden Ker Uplands and south to Spec Uplands. These results are consistent with previous findings published on this group [61].

Climatic and vegetation variables used in the Maxent species distribution models were ranked according to their relative contributions to the model. The three most important environmental predictors of each species’ distribution and their relative percentage contribution to each model are presented in Figures 2–6. Maximum temperature of the warmest period was most frequently the most important factor contributing to the SDMs, including those of all five widespread species were most highly correlated with the maximum temperature of the warmest period. Precipitation variables contributed most to explaining distribution patterns of many species restricted to the central Wet Tropics and vegetation type made the most significant contribution to the distributions of five northern, three central and three southern species.

### Species Richness and Endemism

**Figure 8:**
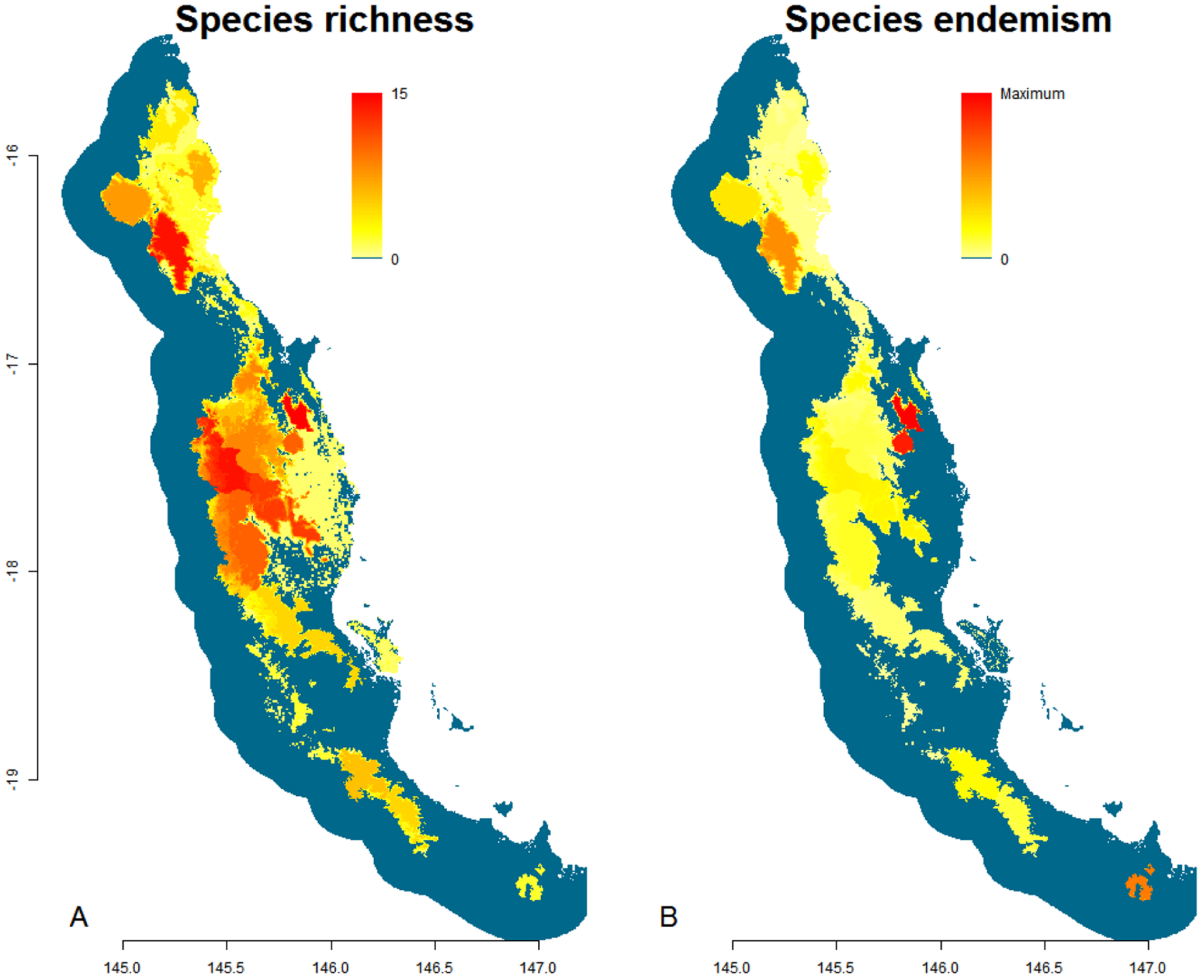
Models of flightless ground beetle species richness and endemism in the Wet Tropics bioregion. **A.** Model of the species richness, calculated by the cumulative total of the modelled distributions of 43 flightless ground beetles within the Wet Tropics. Richness is indicated in a gradient from yellow (less rich) to red (highly rich). Blue areas do not contain distributions of any species. **B.** Model of the species richness weighted for endemism. The modelled distributions of the 43 flightless ground beetles were inversely weighted by range size and summed together. Each subregion is weighted whereby yellow areas contain proportionally few endemic species and red areas display a high proportion of endemics. Blue regions lack any species.

Flightless ground beetles are predicted to be either absent or species poor throughout the vast majority of the Wet Tropics bioregion (Figure 8A). The subregions predicted to contain the highest species richness (15 species) of these beetles included Bellenden Ker Uplands, Carbine Uplands, and Atherton Uplands. Below approximately −18 degrees latitude the predicted species richness is noticeably lower than in the central and northern sections of the Wet Tropics bioregion.

When weighted for endemism, some subregions displayed high proportions of species with restricted distributions (Figure 8b). Greatest predicted endemicity was evident within the Bellenden Ker Uplands, followed by the Carbine Uplands and the southern mountain range of Mt Elliot (Figure 8B). Of the 43 species modelled, five are predicted to be endemic to Bellenden Ker Uplands, with a sixth species only slightly extending its range outside of these mountains. Five species were located only within the Carbine Uplands with a further five having slightly larger ranges including Windsor Uplands or Finnigan Uplands. As mentioned previously, two species were endemic to Mt Elliot, the southernmost mountain range of the Wet Tropics.

**Figure 9:**
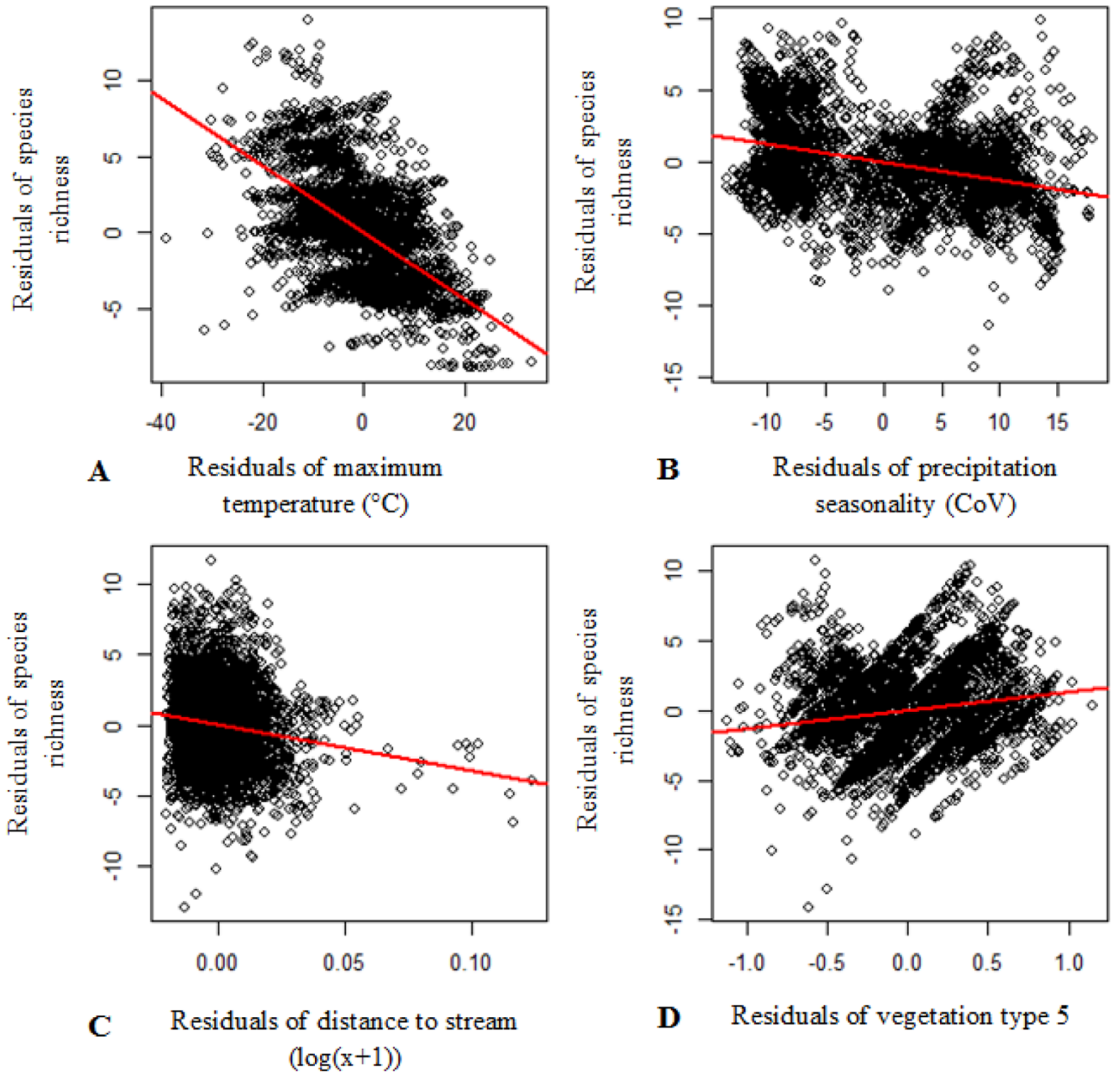
Partial plots of the four environmental factors from the best sub-set multiple linear regression. This analysis identified the combination of environmental factors that best explained patterns of modelled species richness, based on 43 species flightless ground beetles, in the Wet Tropics bioregion. Model Adj. R^2^ = 0.617, s.e. = 2.73, n = 10 000, *P*<0.001. **A**. Residuals of maximum temperature of the warmest period (t value = −45.38, s.e. = 0.004, n = 10 000, Pr(>|t|) <0.001), **B**. residuals of precipitation seasonality (t value = −23.43, s.e. = 0.005, n = 10 000, Pr(>|t|) <0.001), **C**. residuals of distance to stream (t value = −9.6, s.e. = 3.3, n = 10 000, Pr(>|t|) <0.001), **D**. residuals of vegetation type 5 (t value = 10.94, s.e. = 0.12, n = 10 000, Pr(>|t|) <0.001).

The species richness of flightless ground beetles was explained best by a combination of four variables: maximum temperature of the warmest period, precipitation seasonality, distance to stream and notophyll vine forest (model Adj. R^2^ = 0.617, s.e. = 2.73, n = 10 000, *P*<0.001; Table 1). Partial plots of these four environmental variables display significant linear correlations with species richness (Figure 9).

**Table 1:** Best sub-set regression models explaining modelled species richness of flightless ground beetles in the Wet Tropics.

*P* value	Adj. R^2^	Std err.	BIC	Model			
<0.001	0.556	2.94	−8054	max. temp.			
<0.001	0.592	2.82	−8868	max. temp.	precip. s.		
<0.001	0.609	2.76	−9289	max. temp.	precip. s.	stream dist.	
<0.001	0.617	2.73	−9490	max. temp.	precip. s.	stream dist.	veg type 5 (n. v. f.)

Adj. R^2^ = Adjusted R^2^, Std err = standard error, BIC = Bayesian information criterion, max. temp. = maximum temperature of the warmest period, precip. s. = precipitation seasonality, stream dist. = distance to the nearest stream (logx+1), veg type 5 (n. v. f.) = vegetation type 5 (notophyll vine forest).

**Figure 10:**
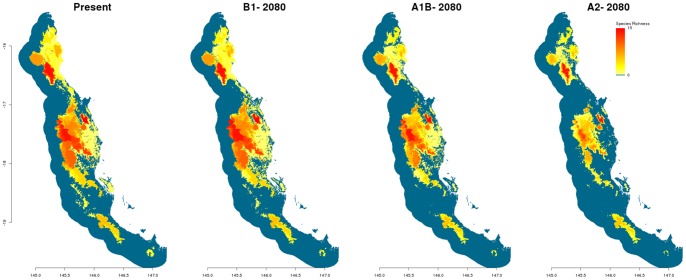
Models of species richness, both present day and future, of flightless ground beetles (n = 43) in the Wet Tropics bioregion. Future models are of the year 2080, for three different emission scenarios (B1, A1B and A2). Richness is indicated in a gradient from yellow (less rich) to red (highly rich). Blue areas are not occupied by any species.

Our modelling suggests that for all emission scenarios, by 2080 there will be substantial declines in the distributions of almost all 43 flightless ground beetles (Appendix S1–S6) and associated declines in the species richness of this group throughout the Wet Topics (Figure 10). As scenarios increase in severity from B1 to A2 they are accompanied by greater losses in species richness. Range contractions are also clearly visible as lower elevations become unsuitable and connectivity between high elevational refugia decreases. However, the refugia of the Carbine Uplands, Bellenden Ker Uplands and Atherton Uplands still maintain relatively high species richness, even under the most severe scenario (A2).

All of the species modelled showed range size contractions (Table 2). The five most-widespread species are projected to contract their range by only 14–31% of their current size (Table 2). Three species, *Notonomus* NQ1, *Pamborus elegans* and *Trichosternus mutatus*, are predicted to go extinct by 2080 under the SRES A2 emission scenario (Table 2). *Notonomus* NQ1 and *Pamborus elegans* are both endemic to Windsor Uplands in the north-west of the Wet Tropics bioregion. *Trichosternus mutatus* is located in the western parts of the central Wet Tropics and is unlikely to be unable to disperse into high refugia such as the Bellenden Ker Uplands. In contrast, the five species endemic to the Bellenden Ker Uplands and Mount Bartle Frere (*Trichosternus fax*, *Mecyclothorax storeyi*, *Notonomus montellus*, *Leiradira alticola* and *Trichosternus montorum*) are predicted to occupy 40–50% of their current ranges (Table 2).

**Table 2:** Data associated with individual species projection models.

species	records	AUC	subregion	lowestelevation	2010range	2080range	2080population
*Notonomus* NQ1	10	0.996	1	972	441	0	0
*Pamborus elegans*	3	0.998	1	1089	390	0	0
*Trichosternus mutatus*	10	0.987	1	769	1399	0	0
*Lecanomerus niger*	32	0.972	3	352	2229	80	1
*Mecyclothorax inflatus*	6	0.996	1	720	847	91	1
*Nurus rex*	6	1	1	834	93	11	1
*Notonomus ellioti*	5	1	1	834	95	15	2
*Notonomus masculinus*	26	0.98	2	598	2097	24	2
*Pamborus euopacus*	68	0.986	3	719	1152	220	2
*Setalis rubripes*	10	0.967	2	686	3203	188	2
*Lacordairia* NQ2	6	0.991	3	772	2541	440	3
*Leiradira alternans*	27	0.964	2	617	2459	449	3
*Notonomus dimorphicus*	9	0.984	1	944	451	120	3
*Pamborus punctatus*	19	0.991	2	769	1317	197	3
*Trichosternus frater*	24	0.973	5	605	1769	245	3
*Castelnaudia setosiceps*	34	0.964	4	101	4260	720	4
*Notonomus flos*	19	0.98	1	829	383	100	4
*Coptocarpus* NQ1	10	0.982	2	608	1072	299	5
*Habutarus nitidicollis*	7	0.998	1	946	420	83	5
*Mystropomus regularis*	68	0.96	7	239	3652	544	5
*Notonomus saepistriatus*	10	0.973	2	685	1661	449	6
*Leiradira* NQ2	16	0.988	2	944	610	190	7
*Mecyclothorax lewisensis*	22	0.995	2	693	564	202	7
*Laccopterum* NQ1	7	0.973	2	711	1380	312	8
*Loxogenius opacipennis*	29	0.991	4	280	2308	429	8
*Notonomus spurgeoni*	46	0.99	3	601	1134	444	8
*Trichosternus fax*	11	0.999	1	1024	184	76	9
*Notonomus doddi*	33	0.975	7	15	3219	998	10
*Pamborus tropicus*	79	0.94	11	24	6531	1594	10
*Leiradira opacistriatus*	26	0.981	5	145	2323	709	14
*Mecyclothorax storeyi*	15	0.999	1	987	204	95	14
*Notonomus montellus*	19	0.999	1	1005	216	105	14
*Leiradira alticola*	15	0.999	1	1005	218	108	15
*Notonomus montorum*	27	0.998	3	781	393	139	15
*Coptocarpus philipi*	7	0.964	2	693	2670	1128	16
*Castelnaudia obscuripennis*	92	0.978	5	19	2711	1067	17
*Trichosternus montorum*	22	0.999	1	987	224	115	17
*Trichosternus soror*	24	0.988	2	581	2150	1008	19
*Castelnaudia spec*	14	0.993	2	640	719	414	26
*Trichosternus nudipes*	16	0.993	2	640	695	459	33
*Leiradira* NQ3	7	0.923	1	387	771	643	55
*Leiradira soror*	8	0.97	3	352	890	792	83
*Castelnaudia* sp.1	5	0.979	1	345	435	395	90

Records represent the number of point locality records used to create the model for each species. AUC is the area under the receiver-operating characteristic curve from each SDM produced. Subregion indicates the number of subregions in which species are known to currently exist and lowest elevation refers to the lowest elevation (m a.s.l.) where the species has been sampled. 2010 range represents the present day modelled range size (km^2^), and 2080 range is the size of the range (km^2^) projected for each species for 2080. 2080 population is the per cent of current population size projected to remain in the year 2080 modelled under emission scenario SRES A2. Species are ranked from the greatest to smallest loss in projected 2080 population size to order species projections from the most to the least vulnerable.

**Figure 11:**
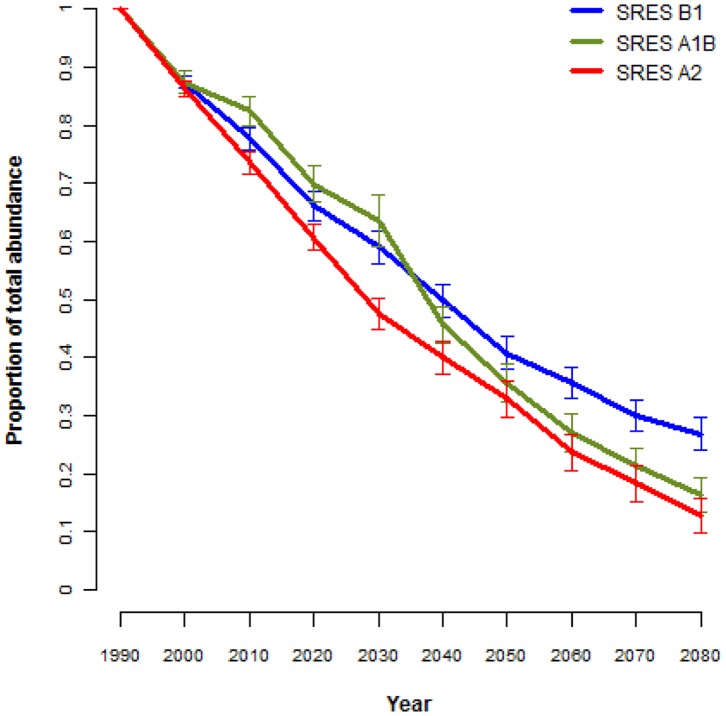
Average predicted proportional change for the total population of flightless ground beetle species (n = 43) in the Wet Tropics. These projections use three emission scenarios (B1, A1B and A2) from the Special Report on Emissions Scenarios (SRES) and eight GCMs. Standard error bars are displayed representing variation between species responses. For individual species projections see Appendix S1–S6.

Total abundance of all flightless ground beetles is projected to greatly decrease under all three emission scenarios by the year 2080 (Figure 11; for individual species responses see Appendix S1–S6). To demonstrate that these conclusions aren’t driven only by the species for which small sample sizes were used in the models, projections were also examined using only the eight species for which more than 30 point localities were available. Declines in the abundance of this group of species were equally severe to those shown by the 43 species combined (Appendix S7). Loss of proportional abundance increases with severity of scenario. Eighty-eight% of species are projected to have less than 20% of their current populations by 2080 (SRES A2; Table 2). Although the five species endemic to the Bellenden Ker Uplands and Mount Bartle Frere are predicted to contract to 40–50% of their current ranges, much of their distributions will be relatively unsuitable, leading to much more dramatic predicted declines in their abundances to 9–17% of their current population sizes (Table 2).

**Figure 12:**
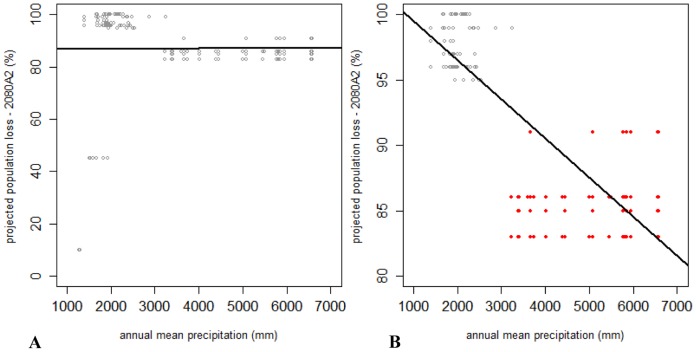
Correlation between projected proportional population loss of subregionally endemic flightless ground beetle species and precipitation. **A.** Correlation of projected proportional population loss by 2080 under SRES A2 and current annual mean precipitation experienced at point localities for all 16 subregional endemic flightless ground beetle species (Adj. R^2^ = 0, res. s.e. = 17, n = 167, *P* = NS). **B.** Correlation of projected proportional population loss by 2080 under SRES A2 and current annual mean precipitation experienced at point localities for the 14 most vulnerable endemics (i.e. projected population loss>80%; Adj. R^2^ = 0.66, res. s.e. = 3.8, n = 146, *P*<0.001). Data pertaining to the five species restricted to the Bellenden Ker Uplands (*Trichosternus fax*, *Mecyclothorax storeyi*, *Notonomus montellus*, *Leiradira alticola* & *Trichosternus montorum*) are indicated in red.

For sixteen species endemic to a particular subregion we attempted to correlate their projected population declines with environmental factors. Two of these, *Leiradira* NQ3 and *Castelnaudia* sp.1, occur at elevations below 700 m a.s.l. (Table 2) and as a consequence were projected to be least vulnerable to climate change impacts (losing 45% and 10% their current populations respectively by the year 2080 under SRES A2; Figure 12A). The remaining 14 species were projected to decline by more than 80% of their current populations (year 2080, SRES A2). Annual mean precipitation was the environmental factor that best explained variance in the projected losses of total abundance for these 14 species (year 2080, SRES A2; model Adj. R^2^ = 0.664, s.e. = 0.037, n = 155, *P*<0.001).

Of these 14 vulnerable subregional endemics, the five restricted to the Bellenden Ker Uplands (*Trichosternus fax*, *Mecyclothorax storeyi*, *Notonomus montellus*, *Leiradira alticola* & *Trichosternus montorum*) were predicted to experience the smallest population declines (Figure 12B). The remaining nine species, endemic to subregions that generally experience less annual precipitation than the Bellenden Ker Uplands, were predicted to lose 95% or more of their current population size (Figure12B). A Fisher test between annual mean precipitation values and population size reductions for all 16 endemic species confirmed a positive association between drier habitats (annual precipitation ≤3,000 mm) and projected population losses for 2080 (SRES A2, Table 3).

**Table 3:** Fisher test of the association of occupation of drier habitats and substantial future population declines for all 16 subregional endemic flightless ground beetles.

		wetter	drier		
extinction	lower	5	2	<95%	population
risk	higher	0	9		reduction

This test determines a positive association between drier (annual precipitation ≤3,000 mm) areas and greater vulnerability to substantial population declines by 2080 (≥95% of current modelled total abundance) as a result of climate change (P value <0.005).

## Discussion

### Links between Environmental Correlates and Distribution and Species Richness Patterns

This study is the first to link distributional and richness patterns of a significant group of flightless invertebrate taxa in the Wet Tropics to climatic factors. Many flightless insects, including carabid beetles, have small upland ranges throughout the Wet Tropics [27], [62]. Yeates *et al.* (2002) investigated the endemicity of flightless insect on both regional and subregional scales. They found that these insects display much greater levels of endemism, at both scales, than Wet Tropics vertebrates. Such highly restricted ranges are thought to have resulted from tropical ground beetles originally colonising lowland regions, then dispersing upwards into montane habitats [63]. Over time, living within relatively stable environmental conditions at high elevations, the beetles no longer needed to disperse long distances and the ability to fly was selected against [63]. Progressively, as cool wet rainforests became restricted to upland habitats in the Wet Tropics so to would have these beetles with such poor dispersal abilities. This scenario is typical of that presented by Ohlemüller *et al.* (2008) where a high proportion of Western Hemisphere birds and European butterflies with small ranges were found in areas that are higher and colder than surrounding habitats. They suggested that such regions may be interglacial refugia, which have receded over time, and where species adapted to cold conditions are able to persist during hotter interglacial periods [64]. If ground beetles in the Wet Tropics were exposed to the above scenario then this would explain why they are so intimately linked to environmental conditions which are commonly found at high elevations in this region.

In the Wet Tropics bioregion, flightless ground beetles are generally confined to, and display highest richness in, cool, wet, stable upland habitats. The modelled distributions of most species are highly restricted with the highest endemism displayed in the Bellenden Ker Uplands. Maximum temperature of the warmest period was most often the environmental factor that was correlated best with individual distribution models. These beetles’ strong links to specific climatic conditions becomes more apparent when examining the environmental factors which were correlated with their species richness throughout the Wet Tropics. Species richness of flightless ground beetles is higher where the rainforest has low maximum temperatures, and rainfall that is more evenly spread throughout the year. This is similar to findings regarding another mountain top taxon in this region, the microhylid frog, whereby high diversity of this group has been linked to consistent levels of moisture throughout the year [65]. Furthermore, distance to the nearest stream negatively correlated, and the presence of notophyll vine forest positively correlated, with the richness of flightless ground beetles. The combination of these environmental correlates defines upland habitats characterised by cool, consistently wet, rainforest. In the Wet Tropics, this habitat type is predominantly confined to small mountain-top areas in and above the cloud cap, which equates to areas above approximately 1,000 m a.s.l.

Historical stability is thought to have had an important influence on current species richness patterns of taxa with low dispersal abilities that are endemic to the Wet Tropics [18]. Subregions determined to have had the greatest historical stability in the Wet Tropics (Bellenden Ker Uplands and Carbine Uplands) are generally those that support the most species of both vertebrate and insect taxa, including flightless ground beetles as demonstrated in this study [18], [62], [66], [67]. Upland rainforest, which is associated with high species richness of flightless ground beetles, has been shown to be highly stable in the Wet Tropics during the late Quaternary [66]. The time-stability hypothesis postulates that stable areas enable species to evolve at a higher rate than habitats characterised by instability [19], [21]. Previous work has implicated historical stability to be an important factor contributing to high richness of flightless invertebrate taxa throughout the Wet Tropics [62] and our findings add weight to such claims.

### Future Projections or Distributions, Species Richness and Population Size

Flightless ground beetles currently confined to marginal habitats are likely to be among the species most vulnerable to climate change impacts in the Wet Tropics. Changes in the distributions flightless ground beetles due to climate change are characterised by upwards range shifts and accompanying range contractions. Worldwide, tropical montane species are expected to undergo upwards shifts in distributions [68]–[70] as are Wet Tropics species, including vertebrates [71], [72], schizophoran flies [40] and dung beetles (Aristophanous, unpublished data).

The severity of Wet Tropic flightless ground beetle range contractions differs between subregions depending on their local climatic conditions, particularly their rainfall patterns. The two highest mountains in the Wet Tropics, Mt Bellenden Ker and Mt Bartle Frere, situated within the Bellenden Ker Uplands, display not only the lowest temperatures, but also the greatest rainfall in the region [36]. As discussed previously, flightless ground beetles are mainly restricted to, and display highest richness in, cool, wet, stable upland habitats. Such habitat is common throughout the Bellenden Ker Uplands and these mountains, therefore, offer the greatest amount of buffering of climate change impacts for flightless ground beetles. In contrast, the most vulnerable subregionally endemic ground beetles are confined to mountains outside of the Bellenden Ker Uplands that are best characterised as currently receiving lower precipitation. Species endemic to the drier mountain ranges, such as the Windsor Uplands and Elliot Uplands, are projected to be extinct by 2080 or reduced to only 1–2% of their current population sizes. This heighted vulnerability of subregionally endemic species in marginal habitats is supported by a positive association between drier habitats and higher projected population losses under the most severe emission scenario in 2080.

Projected distributional contractions of flightless ground beetles in the Wet Tropics negatively affect both the predicted species richness and abundance of this group. As richness projections are directly related to co-located distributional patterns, range contractions resulted in general declines of species richness, especially within the central Atherton Uplands for the most severe scenarios. However, the most alarming results concern the projected impacts of these range contractions on population sizes. The abundance of flightless ground beetles in the Wet Tropics is expected to dramatically decline by the year 2080 due to climate change impacts. Almost 90% of the 43 species examined here, are projected to lose 80% or more of their current populations by 2080 under the most severe emissions scenario with three species predicted to go extinct. Furthermore, dramatic declines occur under all emissions scenarios, reinforcing the sensitivity of this group to even mild levels of climate change. All species are projected to experience population reductions by 2080 with only three species projected to maintain greater than 50% of their current population sizes under the most severe scenario (A2).

These dramatic projected reductions of flightless ground beetle populations suggest that this group is among the most vulnerable to climate change impacts throughout the Wet Tropics. However, there have been very few attempts to quantify likely impacts of climate change on invertebrates in the Wet Tropics. To date only schizophoran flies have been examined in any detail with substantial declines in species richness predicted to occur with an increase in mean temperature of 3°C [40]. Amongst vertebrates, climate change is projected to lead to severe declines for up to 74% of regionally endemic birds in the Wet Tropics [23], [72], [73]. Furthermore, microhylid frogs are predicted to undergo dramatic reductions in population sizes throughout the Wet Tropics. Shoo [71] projected population losses of microhylid frogs in relation to temperature increases in this region. Of the six frog species modelled in relation to a four degree increase, four were projected to be extinct, one almost extinct and the last had reduced in population size by over 80% [71]. This study confirms the prediction of Williams *et al.* (2008a) that insects of low vagility in the Wet Tropics will be similarly vulnerable to climate change impacts as regionally endemic vertebrates.

Flightless ground beetles constitute a large component, both in terms of abundance and body size, of the fauna of predatory insects found at high elevations in the Wet Tropics [27]. Consequently, the substantial reduction in carabid beetle populations projected to result from climate change has the potential to alter the community dynamics of the ground fauna. As leaf litter invertebrates perform vital roles in nutrient cycling [74], suppression of this major predatory group could even potentially alter this ecosystem function. Population declines of flightless ground beetles may also have impacts at higher trophic levels as they are thought to be an important food source for many vertebrates, including mammals [28], [75].

Future precipitation changes are notoriously difficult to predict [76] and often limit projections of species distributions with climate change scenarios. However, this study has demonstrated strong links between ground beetle distributions, abundance and richness with precipitation levels. Therefore, it is worthwhile noting that any increase in dry season severity, rise in the level of cloud caps and subsequent reductions in cloud stripping, or other reductions in rainfall would be expected to further compromise the survival of flightless ground beetles in the Wet Tropics. Additionally, although projected changes in vegetation were not incorporated into our future scenario models, the results of Hilbert [53] suggest that the extent of highland rainforests may decrease by 50% with only one degree of warming. In light of the relationships between ground beetle distributions and upland rainforest in the Wet Tropics, such dramatic contractions of this habitat type are likely to exacerbate future reductions in ground beetles distributions, richness and population size.

## Conclusion

This study is the first to model changes in the distribution, richness and abundance of beetles under future climate change scenarios. We demonstrate that current distributions and richness of flightless ground beetles in the Wet Tropics are best correlated with high elevations, characterised by cool, moist and stable environmental conditions. These findings support the notion that differences in historical climatic stability between subregions have substantially influenced current biodiversity patterns.

Flightless ground beetles restricted to more marginal mountain ranges are projected to be the most vulnerable to climate change impacts in the Wet Tropics. Future ranges are projected to contract as distributions shift upwards and subsequently species richness is expected to decline. Ultimately however, the greatest impact on flightless ground beetles is the extreme reductions in abundance with the vast majority of species projected to lose 80% or more of their current population size.

These analyses not only describe the strong links flightless ground beetles have to climatic conditions, but also stress the negative impacts expected from future changes in these climatic correlates. The high level of sensitivity noted in this group implies that other low vagility invertebrate taxa, the vast majority of which remain unstudied, may also be similarly threatened by climate change. Future research must address this lack of understanding if climate change impacts on mountain-top ecosystems are to be comprehensively understood.

## Supporting Information

Appendix S1 Projected changes in the proportion of total current population for 8 of 43 flightless ground beetle species in the Wet Tropics by the year 2080. Species displayed are: *Castelnaudia obscuripennis*, *Castelnaudia setosiceps*, *Castelnaudia sp.1*, *Castelnaudia spec*, *Coptocarpus NQ1*, *Coptocarpus philipi*, *Habutarus nitidicollis* and *Laccopterum NQ1*. Projections use three emission scenarios from the SRES (B1, A1B and A2) and eight GCMs. Error bars represent variation between model outputs.
(TIF)

Appendix S2 Projected changes in the proportion of total current population for 8 of 43 flightless ground beetle species in the Wet Tropics by the year 2080. Species displayed are: *Lacordairia NQ2*, *Lecanomerus niger*, *Leiradira alternans*, *Leiradira alticola*, *Leiradira NQ2*, *Leiradira NQ3*, *Leiradira opacistriatus* and *Leiradira soror.* Projections use three emission scenarios from the SRES (B1, A1B and A2) and eight GCMs. Error bars represent variation between model outputs.
(TIF)

Appendix S3 Projected changes in the proportion of total current population for 8 of 43 flightless ground beetle species in the Wet Tropics by the year 2080. Species displayed are: *Loxogenius opacipennis*, *Mecyclothorax inflatus*, *Mecyclothorax lewisensis*, *Mecyclothorax storey*, *Mystropomus regularis*, *Notonomus dimorphicus*, *Notonomus doddi* and *Notonomus ellioti*. Projections use three emission scenarios from the SRES (B1, A1B and A2) and eight GCMs. Error bars represent variation between model outputs.
(TIF)

Appendix S4 Projected changes in the proportion of total current population for 8 of 43 flightless ground beetle species in the Wet Tropics by the year 2080. Species displayed are: *Notonomus flos*, *Notonomus masculinus*, *Notonomus montellus*, *Notonomus montorum*, *Notonomus NQ1*, *Notonomus saepistriatus*, *Notonomus spurgeoni* and *Nurus rex*. Projections use three emission scenarios from the SRES (B1, A1B and A2) and eight GCMs. Error bars represent variation between model outputs.
(TIF)

Appendix S5 Projected changes in the proportion of total current population for 8 of 43 flightless ground beetle species in the Wet Tropics by the year 2080. Species displayed are: *Pamborus elegans*, *Pamborus euopacus*, *Pamborus punctatus*, *Pamborus tropicus*, *Setalis rubripes*, *Trichosternus fax*, *Trichosternus frater* and *Trichosternus montorum*. Projections use three emission scenarios from the SRES (B1, A1B and A2) and eight GCMs. Error bars represent variation between model outputs.
(TIF)

Appendix S6 Projected changes in the proportion of total current population for 3 of 43 flightless ground beetle species in the Wet Tropics by the year 2080. Species displayed are: *Trichosternus mutatus*, *Trichosternus nudipes* and *Trichosternus soror*. Projections use three emission scenarios from the SRES (B1, A1B and A2) and eight GCMs. Error bars represent variation between model outputs.
(TIF)

Appendix S7 Projected changes in the proportion of total current population for the eight flightless ground beetle species in the Wet Tropics for which more than 30 point locality records were obtained. Species included are: *Lecanomerus niger, Pamborus euopacus, Castelnaudia setosiceps, Mystropomus regularis, Notonomus spurgeoni, Notonomus doddi, Pamborus tropicus and Castelnaudia obscuripennis*. Projections use three emission scenarios from the SRES (B1, A1B and A2) and eight GCMs. Error bars represent variation between model outputs.
(TIF)
