# Supplementary figures and images for: Projected Distributions and Diversity of Flightless Ground Beetles within the Australian Wet Tropics and Their Environmental Correlates
Source: PLoS One. 2014 Feb 20;9(2):e88635. doi: 10.1371/journal.pone.0088635 (PMC3930578; doi:10.1371/journal.pone.0088635)

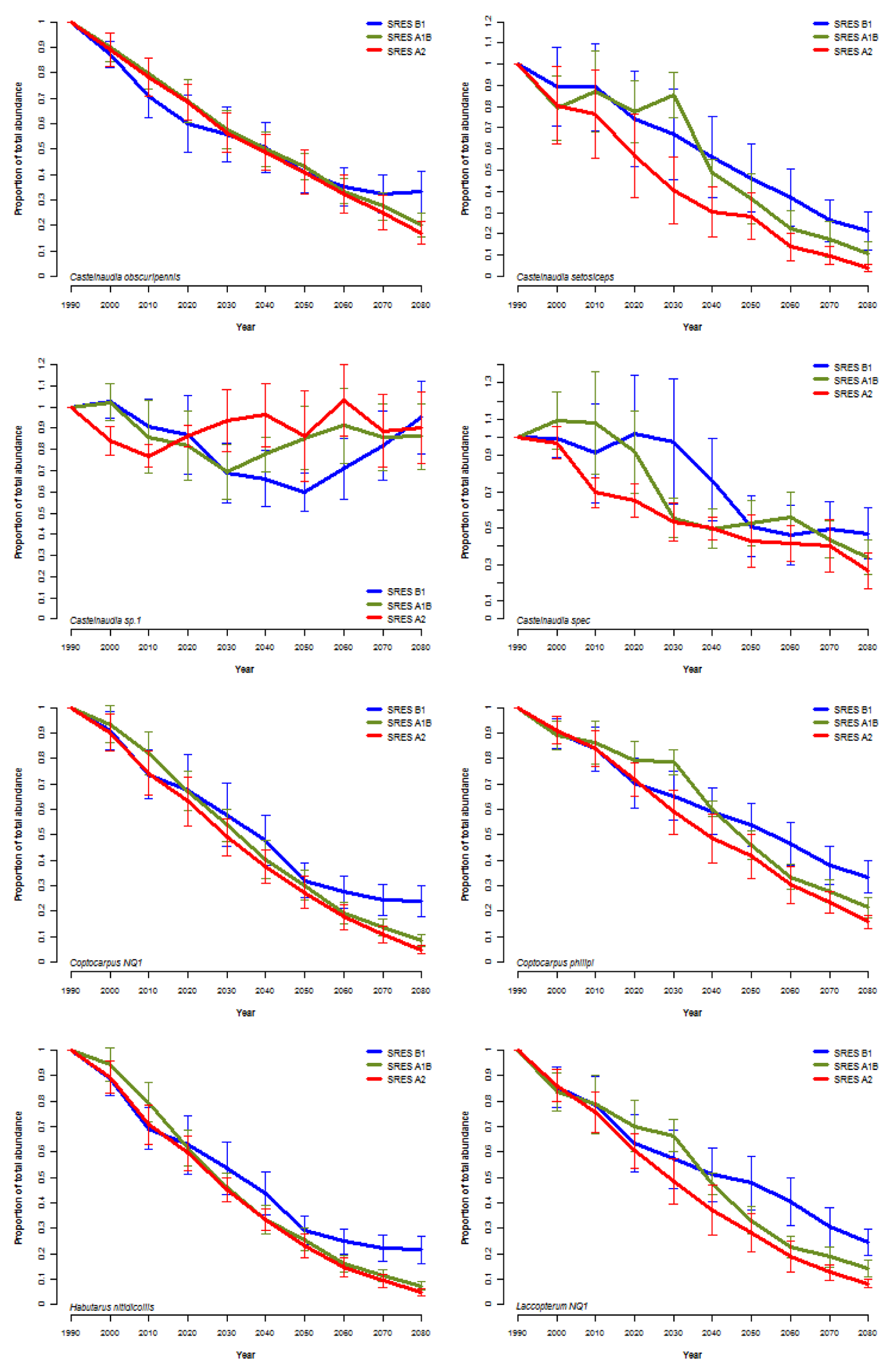

Supplement: Appendix S1 — Projected changes in the proportion of total current population for 8 of 43 flightless ground beetle species in the Wet Tropics by the year 2080. Species displayed are: Castelnaudia obscuripennis, Castelnaudia setosiceps, Castelnaudia sp.1, Castelnaudia spec, Coptocarpus NQ1, Coptocarpus philipi, Habutarus nitidicollis and Laccopterum NQ1. Projections use three emission scenarios from the SRES (B1, A1B and A2) and eight GCMs. Error bars represent variation between model outputs. (TIF) [file pone.0088635.s001.tif]

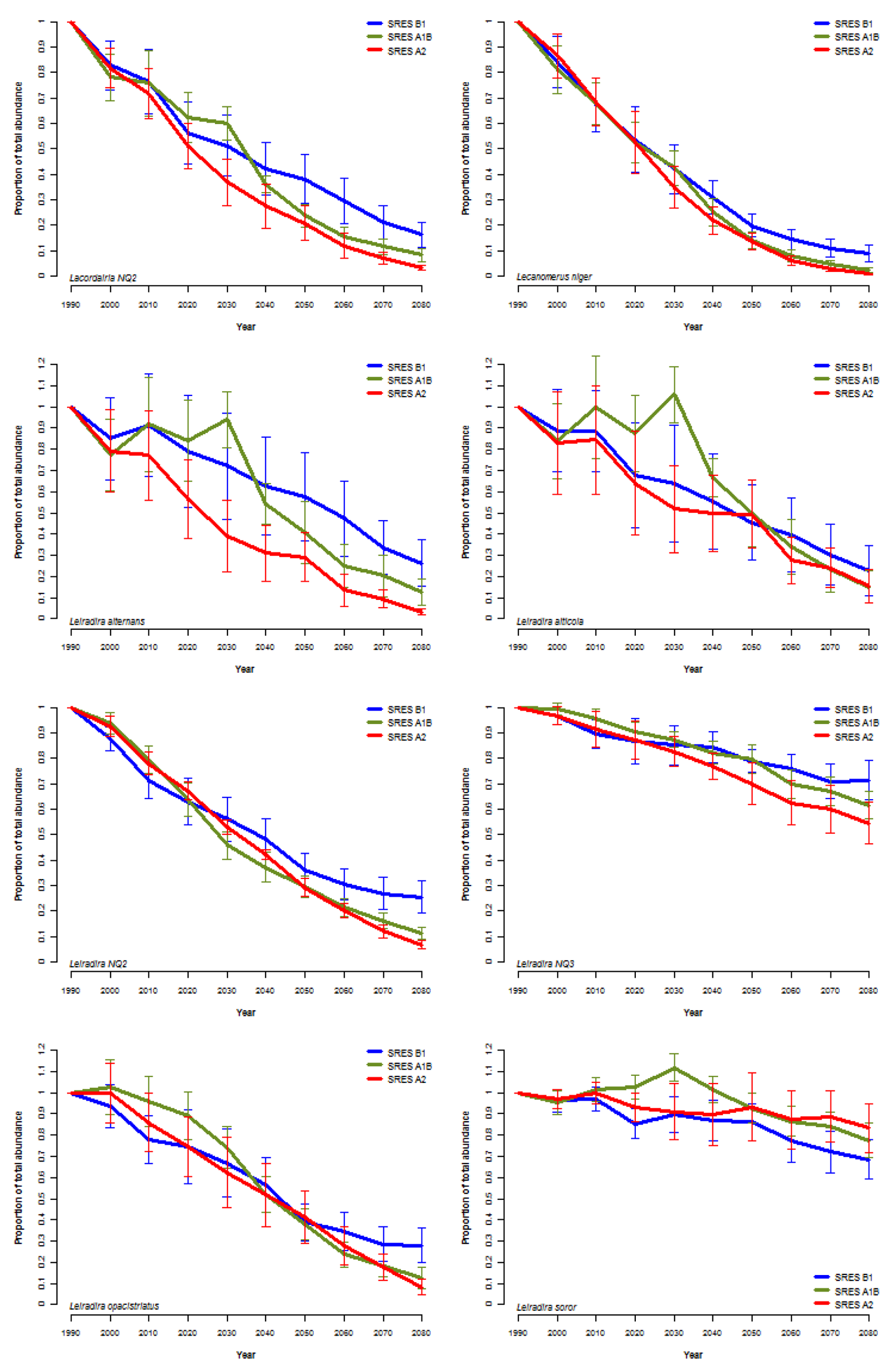

Supplement: Appendix S2 — Projected changes in the proportion of total current population for 8 of 43 flightless ground beetle species in the Wet Tropics by the year 2080. Species displayed are: Lacordairia NQ2, Lecanomerus niger, Leiradira alternans, Leiradira alticola, Leiradira NQ2, Leiradira NQ3, Leiradira opacistriatus and Leiradira soror. Projections use three emission scenarios from the SRES (B1, A1B and A2) and eight GCMs. Error bars represent variation between model outputs. (TIF) [file pone.0088635.s002.tif]

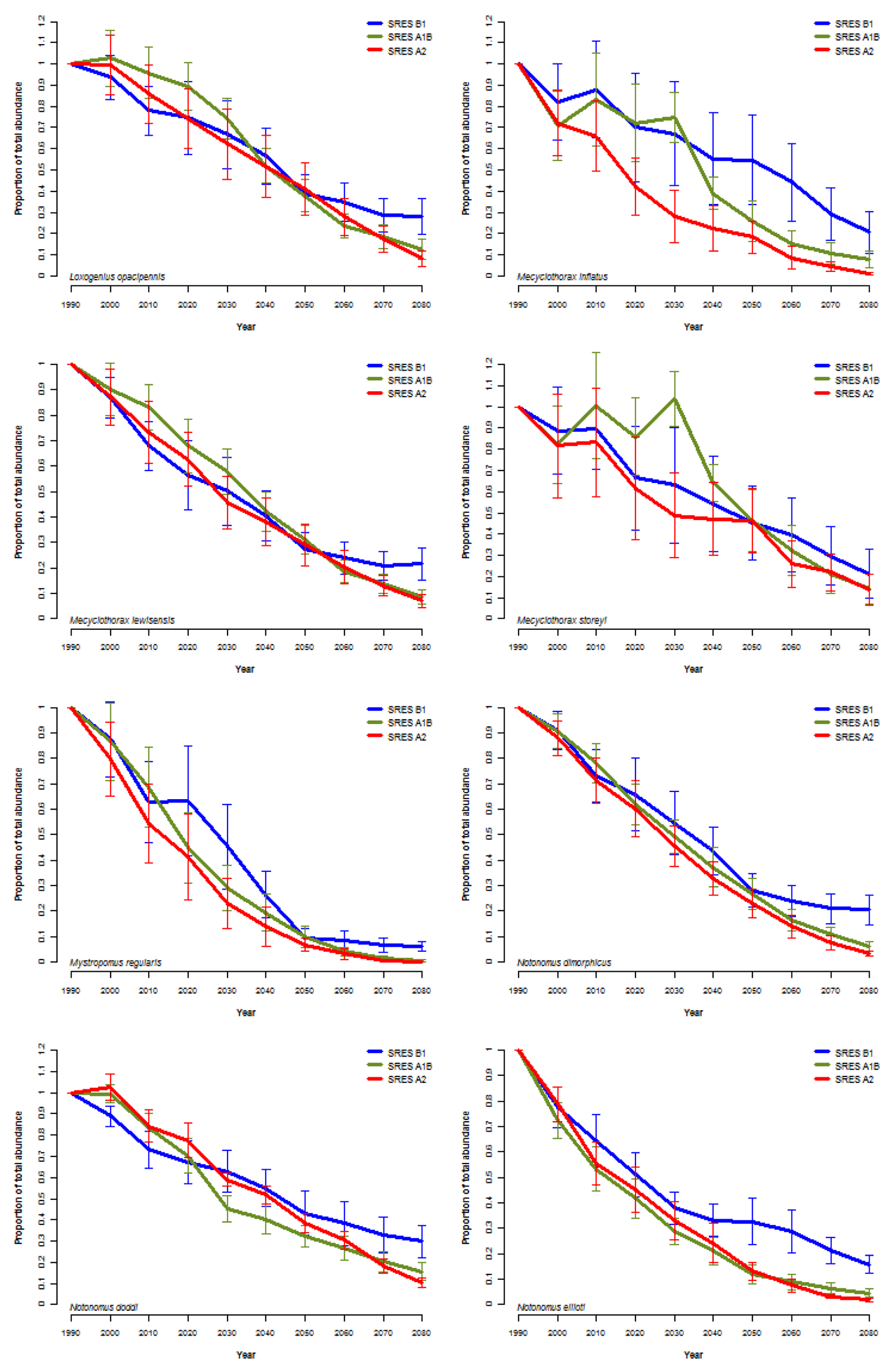

Supplement: Appendix S3 — Projected changes in the proportion of total current population for 8 of 43 flightless ground beetle species in the Wet Tropics by the year 2080. Species displayed are: Loxogenius opacipennis, Mecyclothorax inflatus, Mecyclothorax lewisensis, Mecyclothorax storey, Mystropomus regularis, Notonomus dimorphicus, Notonomus doddi and Notonomus ellioti. Projections use three emission scenarios from the SRES (B1, A1B and A2) and eight GCMs. Error bars represent variation between model outputs. (TIF) [file pone.0088635.s003.tif]

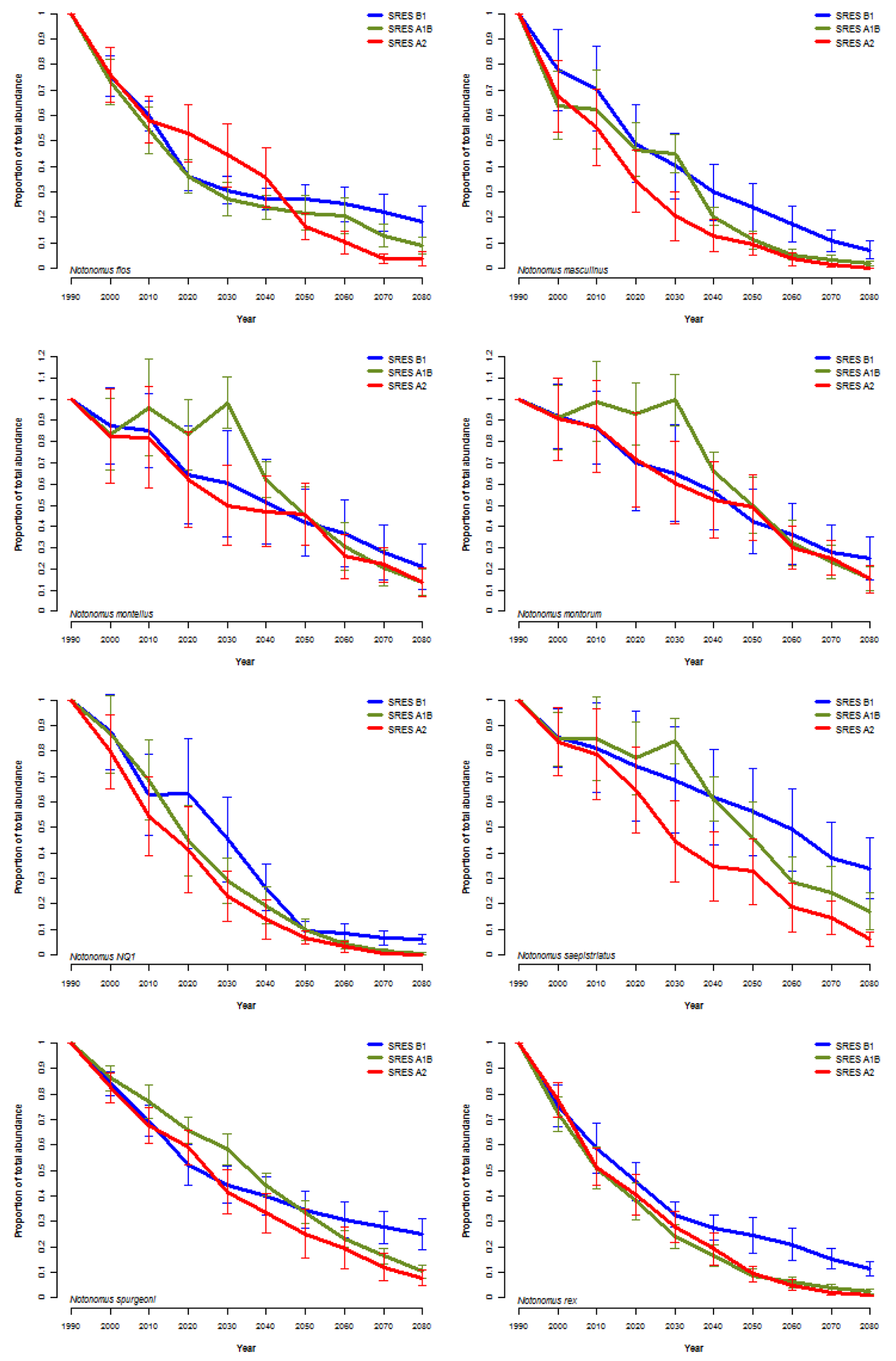

Supplement: Appendix S4 — Projected changes in the proportion of total current population for 8 of 43 flightless ground beetle species in the Wet Tropics by the year 2080. Species displayed are: Notonomus flos, Notonomus masculinus, Notonomus montellus, Notonomus montorum, Notonomus NQ1, Notonomus saepistriatus, Notonomus spurgeoni and Nurus rex. Projections use three emission scenarios from the SRES (B1, A1B and A2) and eight GCMs. Error bars represent variation between model outputs. (TIF) [file pone.0088635.s004.tif]

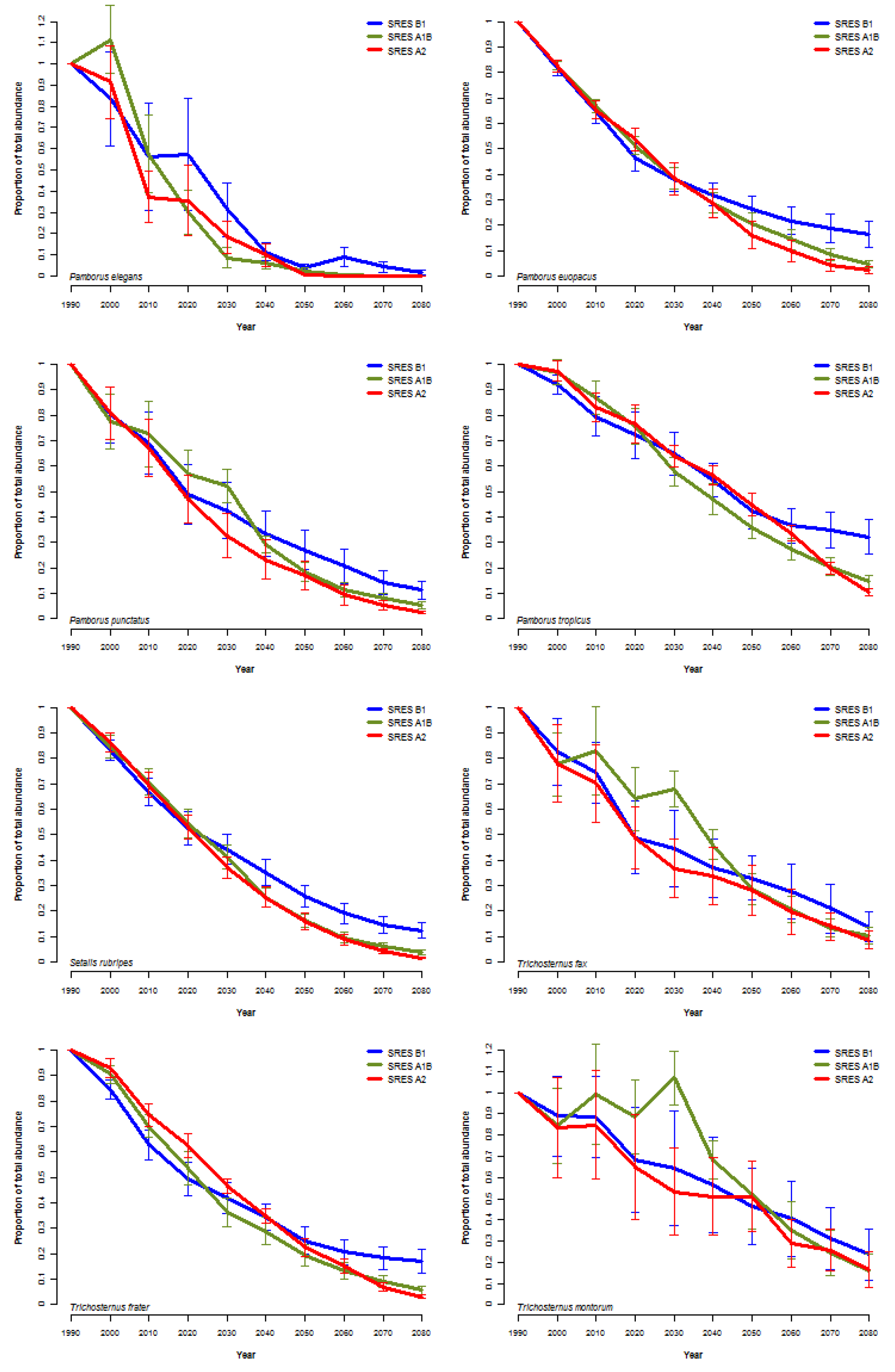

Supplement: Appendix S5 — Projected changes in the proportion of total current population for 8 of 43 flightless ground beetle species in the Wet Tropics by the year 2080. Species displayed are: Pamborus elegans, Pamborus euopacus, Pamborus punctatus, Pamborus tropicus, Setalis rubripes, Trichosternus fax, Trichosternus frater and Trichosternus montorum. Projections use three emission scenarios from the SRES (B1, A1B and A2) and eight GCMs. Error bars represent variation between model outputs. (TIF) [file pone.0088635.s005.tif]

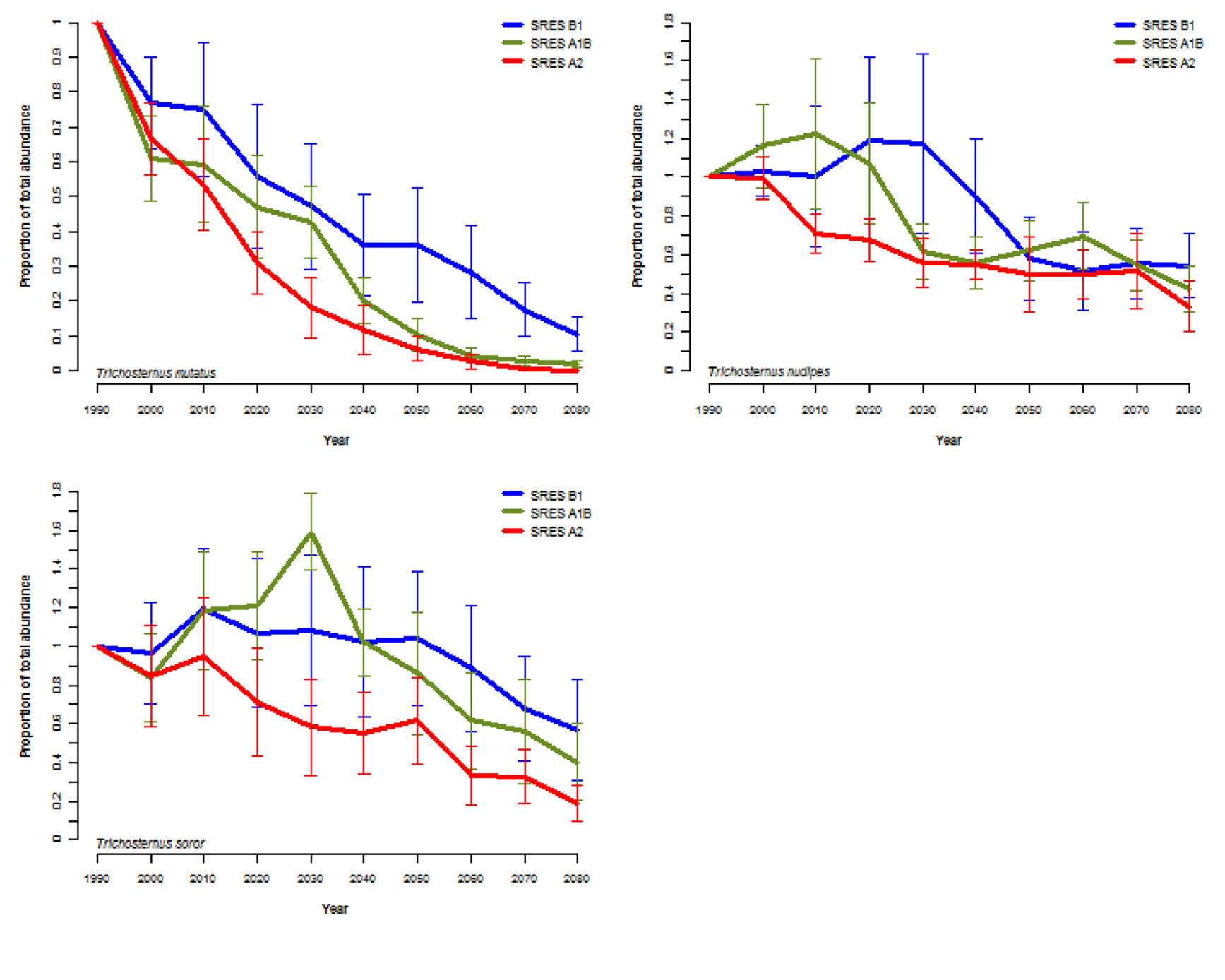

Supplement: Appendix S6 — Projected changes in the proportion of total current population for 3 of 43 flightless ground beetle species in the Wet Tropics by the year 2080. Species displayed are: Trichosternus mutatus, Trichosternus nudipes and Trichosternus soror. Projections use three emission scenarios from the SRES (B1, A1B and A2) and eight GCMs. Error bars represent variation between model outputs. (TIF) [file pone.0088635.s006.tif]

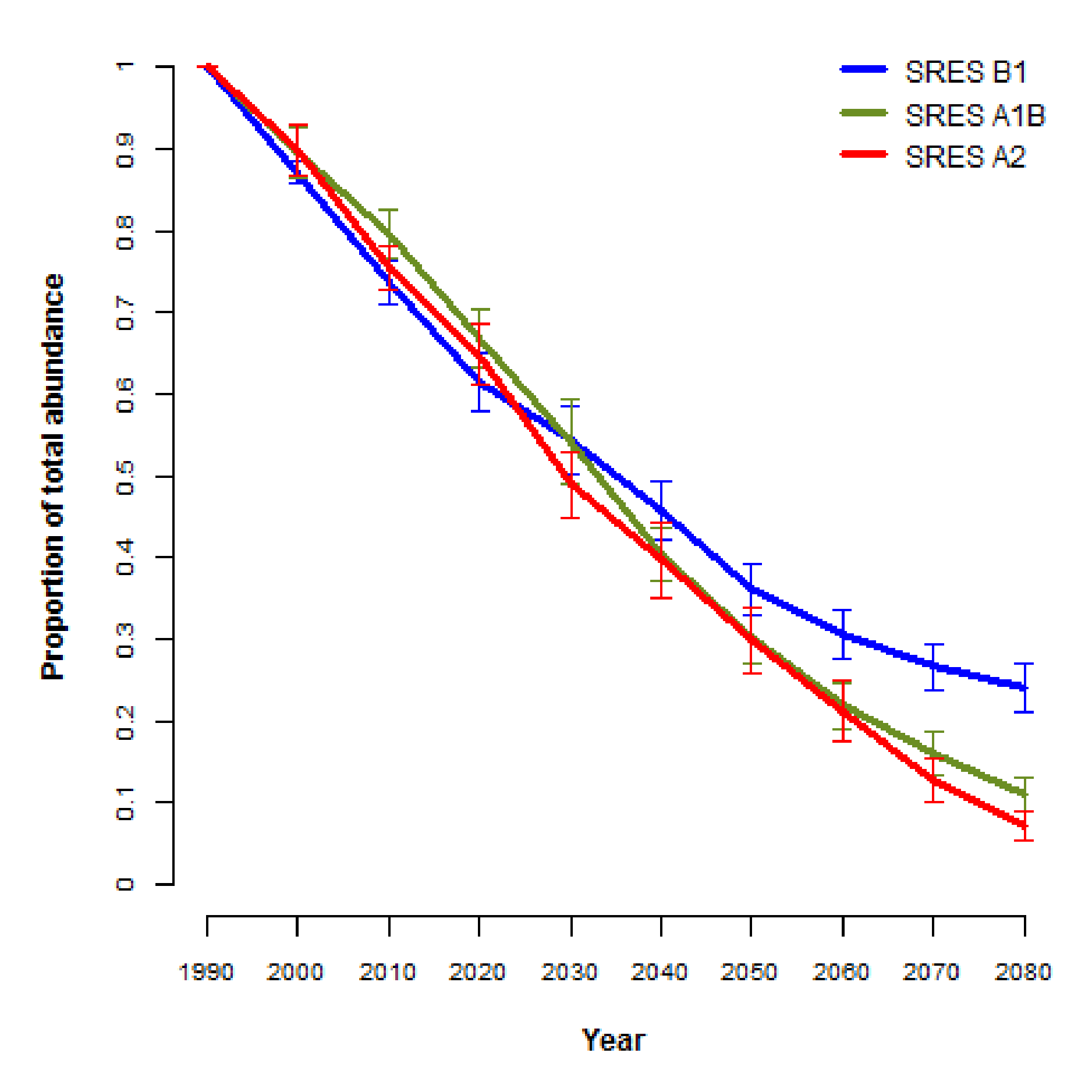

Supplement: Appendix S7 — Projected changes in the proportion of total current population for the eight flightless ground beetle species in the Wet Tropics for which more than 30 point locality records were obtained. Species included are: Lecanomerus niger, Pamborus euopacus, Castelnaudia setosiceps, Mystropomus regularis, Notonomus spurgeoni, Notonomus doddi, Pamborus tropicus and Castelnaudia obscuripennis. Projections use three emission scenarios from the SRES (B1, A1B and A2) and eight GCMs. Error bars represent variation between model outputs. (TIF) [file pone.0088635.s007.tif]
